# Monthly Summary

**Published:** 1875-10

**Authors:** 


### MONTHLY SUMMARY.

---

*Dispensing with Rain.*—As calcium chlorid possesses the property of attracting moisture, and that an object wetted with its aqueous solution will not dry, it has been proposed to water our streets with such a solution in order to avoid dust in summer. Prof Paraf proposes to extend its use to agriculture, and to moisten all kinds of soil with it in dry regions where rain is deficient. He says that its application produces irritation more cheaply than any other method. He found that a piece of land where this is applied will retain its moisture for three days, while a similar piece of land next to it, irrigated with water alone, will dry in a single hour. He claims that this will not only make two blades of grass grow where only one grew before, but render to sandy and desert wastes a luxuriant vegetation, and consequent prosperity. It will have to be found out in how far the presence of an excess of calcium chlorid is beneficial to some plants and injurious to others. We fear the latter, as the presence of sea-water, in which calcium and sodium chlorids are the main ingredients, is injurious to most plants. Thus the low lands in Holland (of which from time to time some are being redeemed from the sea by ditches) remain unproductive until in the course of time the rains and careful drainage have washed out most of the salts left by the sea-water.—*Manufacturer and Builder.*

*Ether vs. Chloroform.*—I have almost discarded chloroform and bichloride of methylene, both in hospital and in private practice, and, for adults, use ether as my sole anæsthetic. The secret of administration is to give ether enough, and to exclude external air until insensibility is produced. We sometimes use chloroform for young children, who generally take it kindly, and we sometimes give a few whiffs of chloroform to render irritable air-passages insensitive to ether, but that is all. The contrast between the effects of the two agents is very striking. An old patient brought deeply under chloroform, generally becomes very pallid and bathed in sweat; but the same patient under ether would have a flushed face, and would be sustained and stimulated instead of being depressed. Chloroform and bichloride of methylene have no advantage over ether, except that they act a little more quickly; and the gain of a few seconds affords no justification for the sacrifice of human life.—*Mr. R. B. Carter.*

The committee appointed by the Royal Medical and Chirugical Society to investigate the effects of chloroform found that "the same failure of respiration, whilst the circulation remains good, almost always betokens a recoverable condition;" but that "the failure of the circulation to any considerable extent always involves extreme peril;" and "after the heart has stopped, recovery is but just possible, and is by no means the usual result of attempts to resuscitate." So that the danger from chloroform is precisely of that kind which it is most hopeless when it occurs. On the other hand, the same committee state that ether vapor stimulates the heart's action, and that the pressure in the vessels is maintained until there has been a manifest failure of the breathing. And Dr. Snow proved that it was impossible to paralyze the heart by ether inhalation. It is this stimulating property which gives to ether its greater safety and enables us to use it not only as an anæsthetic, but as an antagonist to the shcck of an operation. Under the influence of ether. as I have often pointed out, the patient may have a better pulse after than before even a severe operation—(*Mr. W. W. Haward.*)—*Braithwaite's Retrospect.*

---

*Effects of the Inhalation of Chloroform during Parturition, on the Fetus.*—It is stated in foreign jonrnals, that Dr. Sweifel, of Strasburg, submitted the placenta to analysis, by distillation' in a case where chloroform had been inhaled, and obtained chloroform, recognized by several accurate tests. He has also found that the urine of new born children whose mothers had used chloroform reduces Fehling's solution, and has, moreover, observed that the expired air of such children has, for several hours, a well-marked odor of chloroform. Dr. Sweifel, arguing from these facts, concludes that the chloroform inhaled by the mother passes into the circulation of the fœtus.—*Med. & Surg. Reporter.*

